# Amyloidosis of the Heart and Kidney

**DOI:** 10.14797/mdcvj.1150

**Published:** 2022-09-06

**Authors:** Horacio E. Adrogue

**Affiliations:** 1 Division of Nephrology, Transplantation, and Hypertension, Department of Medicine, Houston Methodist Hospital, Houston, Texas, US; 2 Texas A&M Medical School, Houston, Texas, US

**Keywords:** amyloidosis, kidney transplant, heart transplant, stem cell transplant, carpel tunnel syndrome

## Abstract

Amyloidosis encompasses a collection of disorders of pathological protein folding. The extracellular location where these “amyloid fibril” proteins are deposited determines the clinical presentation of the disease. The abnormal architecture of these fibrils makes them insoluble and not easily removed, leading to disruption of normal tissue structure and interference with normal physiology. Amyloidosis of the heart and kidney can be inherited, secondary to unrelated diseases, or due to a plasma cell disorder. This review will focus on immunoglobulin light chain amyloidosis, which is life-threatening and must be diagnosed as early as possible by employing precise and accurate typing to ensure timely and frequently curative therapy.

## Introduction

Amyloidosis type is classified using the following nomenclature.^1^ The “A” signifies the fibril protein, which varies according to the cause of the amyloidosis (called protein A) and is followed by a suffix that is an abbreviation of the name of the precursor protein. Immunoglobulin light chain amyloidosis (AL amyloidosis) is thus due to light chains, which are produced in the bone marrow by a malignant B-cell clone. AA amyloid is associated with chronic inflammatory conditions such as Familial Mediterranean fever, rheumatoid arthritis, systemic lupus erythematosus, and others. Transthyretin amyloidosis (ATTR) is due to a genetic defect in the transthyretin (TTR) protein, while chronic kidney disease patients on long-term hemodialysis can suffer from B2 amyloidosis, which is due to the accumulation of beta 2 microglobulin. Both types can cause symptomatic carpel tunnel syndrome. When these patients undergo carpel tunnel release surgery, it is important that the surgeon send tissue samples of the tenosynovium transverse carpel ligament or facia and test the amyloid type. While dialysis patients are more likely to have beta 2 microglobulin amyloid, it is now commonly recognized that patients with ATTR can present with bilateral carpel tunnel syndrome. A recent prospective cohort study of men ≥ 50 years and woman ≥ 60 years who presented with bilateral carpel tunnel syndrome reported a 10.2% rate of amyloid deposits.^2^ In another study, carpel tunnel syndrome preceded clinical heart failure in ATTR by a mean of 6.1 years.^3^ Bilateral carpel tunnel syndrome should now be considered an early warning signal to occult amyloidosis.

While this article focuses on the cardiac and kidney involvement of AL amyloidosis, almost any organ can be affected, and its nonspecific and varied presentation may lead to late diagnosis. In fact, most patients see three to four physicians before receiving a definitive diagnosis.^4^

AL amyloidosis has an incidence of 4,000 new cases in the United States (US) per year^5^ and a prevalence of 12,000 cases.^6^ ATTRv, a variant that results from gene mutations and has a worldwide prevalence of 10,000 to 40,000 cases,^7^ is due to misfolding of the destabilized TTR protein that is a transporter of retinol and thyroxine. Wild-type ATTR (ATTRwt) results from misfolding of the non-mutated or wild-type TTR protein, previously known as “senile amyloid.” Thirteen percent of a group of elderly patients admitted with heart failure with preserved ejection fraction^8^ were found to have ATTRwt. Additionally, 16% of those who had a transcatheter aortic valve replacement for calcific aortic stenosis were also found to have ARRTwt.^9^ Additionally, a Finnish autopsy report of those over 85 years old reported that 25% of the hearts involved ATTRwt.^10^

## Diagnostic Approach

### Step one: early and accurate diagnosis of origin of amyloid fibril

**Figure 1:**
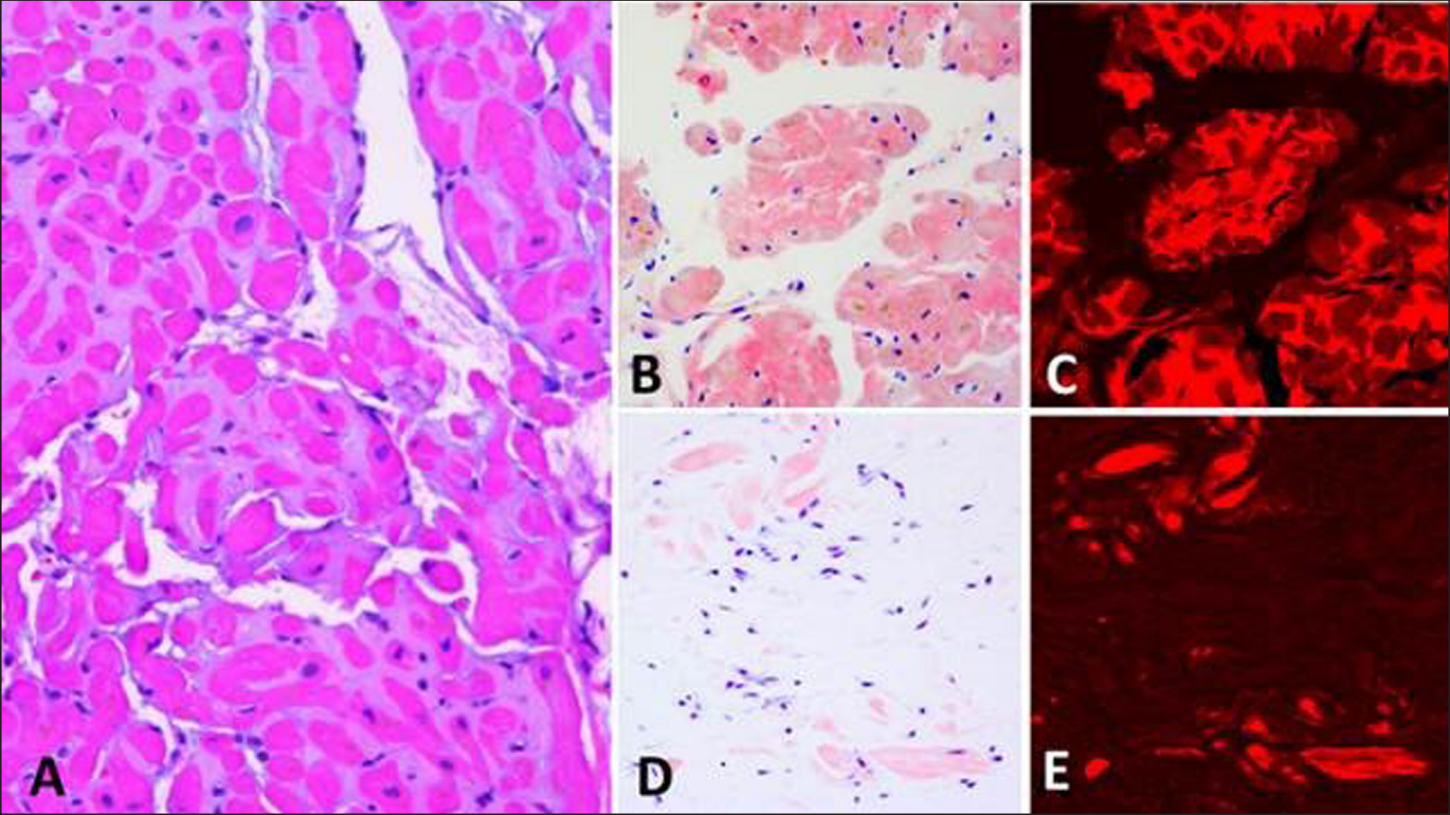
**(A-C)** Cardiac amyloidosis. **(A)** Amyloid appearing as a glassy eosinophilic material surrounding myocardial fibers (Hematoxylin&eosin ×200). **(B, C)** Amyloid displaying a “salmon-pink” color under Congo red stain (×200), which is strongly enhanced under Texas red immunofluorescent examination (×200). **(D, E)** Tenosynovial amyloidosis. A small amount of amyloid not apparent in routine tissue section appearing here as small “salmon-pink” nodules, which are strongly enhanced under Texas red immunofluorescent examination (×200). Even this small amount of amyloid is suitable for mass spectrometric analysis; in this case it shows transthyretin type of amyloid. Transthyretin amyloid identified in tissue removed for carpal tunnel syndrome may signify concurrent or future cardiac amyloidosis of the same chemical type.

Due to the vague nature of symptoms associated with this disease, the median time from initial presentation to diagnosis is 6 to 12 months. A nephrologist may be consulted for protein in the urine,^11^ and more than 50% of patients will present with more than 3 g of protein in the urine. Nevertheless, as little as 250 mg of proteinuria, along with a concerning history, should prompt an evaluation. Frequently, a kidney biopsy is needed to rule out AL amyloidosis.^12^ In cases of amyloid deposits limited to blood vessels, acute kidney failure may be seen without proteinuria.^13^ Heart involvement (Figure 1A, B, C) may be more difficult to diagnose and lead to greater delay in diagnosis. One of the most common complaints from over 80% of patients is fatigue.^14^ Dyspnea on exertion, leg edema, and easy bruising also are frequent complaints.

Macroglossia, occurring in only 10% to 15% of patients, may cause new onset of snoring or obstructive sleep apnea or biting the sides of the tongue. At times, family members may tell the patient that is has become difficult to understand their speech. The patients themselves may experience the sensation that their tongue has become too large for their mouth. The amyloid deposit starts at the base of the tongue, so a cursory look at the tongue may not detect anything initially. When affecting the nervous system, patients may present with imbalance, symmetric painful neuropathy, orthostatic hypotension, urinary retention, early satiety, and occasionally erectile dysfunction. Seated and standing blood pressures, basic metabolic panel, terminal brain natruretic peptide (NT–pro BNP) troponin I or T, alkaline phosphatase, and urine analysis with a spot urine for protein and creatinine are all standard parts of the evaluation.^15^ If neuropathy is present, then electromyography (EMG) and nerve conduction studies are indicated.

Once an affected organ is identified, a biopsy should be done, including personally communicating to the pathologist that there is a suspicion of AL amyloidosis. The sample should always be sent for typing before any therapy is initiated. A combination of a fat pad biopsy and a bone marrow biopsy will secure diagnosis ≥ 90% of cases.^16^ Some patients may have an increased risk of bleeding from acquired factor X deficiency secondary to the adsorption of factor X by amyloid fibrils.^17^ Therefore, a kidney biopsy may not always be done safely. Tissue should be stained for Congo red as well as thioflavin T sulfate alcian blue.^17^ If positive, tissue should be processed to determine the precursor protein by micro dissection or mass spectrometry, as both have high sensitivity and specificity.^18^ Mass spectrometry has replaced immunofluorescence in centers that have the ability to detect M–proteins with light chain glycosylation.

Serum and urine electrophoresis with immunofixation and a serum free light chain assay are the cornerstone of the evaluation of AL amyloidosis. Bone marrow aspiration and biopsy with fluorescence in situ hybridization (FISH) is critical for treatment planning.

It is also important to remember that as kidney function diminishes the ratio of kappa and lambda light chains may be elevated up to 3:1. This is still in keeping with chronic kidney disease and should not increase the pretest probability of having AL amyloidosis.

## Prognosis

Prognosis is heavily dependent on the organ involved and plasma cell clone type. The degree of cardiac involvement is the single most important predictor of both short- and long-term patient survival. Involvement of other systems could preclude solid organ transplant. An important example of a medical relative contraindication for solid organ transplantation is orthostatic hypotension, as its presence is associated with a greatly reduced life expectancy. The nature of the plasma cell clone is also important for long-term survival. A recent paper from the Mayo Clinic was encouraging, showing improvement in the 6-month death rate among newly diagnosed patients with AL amyloidosis: Those diagnosed prior to 2005 had a 37% death rate, which dropped significantly to 25% for those diagnosed after 2005.^19^

### Heart Involvement

The most recent Mayo Clinic 2012 staging system incorporated troponin T, NT-proBNP, and immunoglobulin free light chains (Table 1).^20^ While echocardiography is prognostically important, the significant variability in test performance between centers precludes its ability to be used as a broad diagnostic criterion. Ejection fraction, longitudinal left ventricular strain, and stroke volume index are all examples of echocardiographic measures that have been used to further stratify risk for individual patients at local centers.^21^

**Table 1:** Revised Mayo Clinic staging of AL amyloidosis (with chemotherapy). Scores are calculated by giving 1 point for each of the following: troponin T ≥ 0.025 ng/ml; N-T pro BMP ≥ 1,800 pg/ml; difference between involved and uninvolved serum free light chain levels >180 mg/L.


SCORE	MEDIAN SURVIVAL (MONTHS)

0	94

1	40

2	14

3	6


### Kidney Involvement

**Figure 2:**
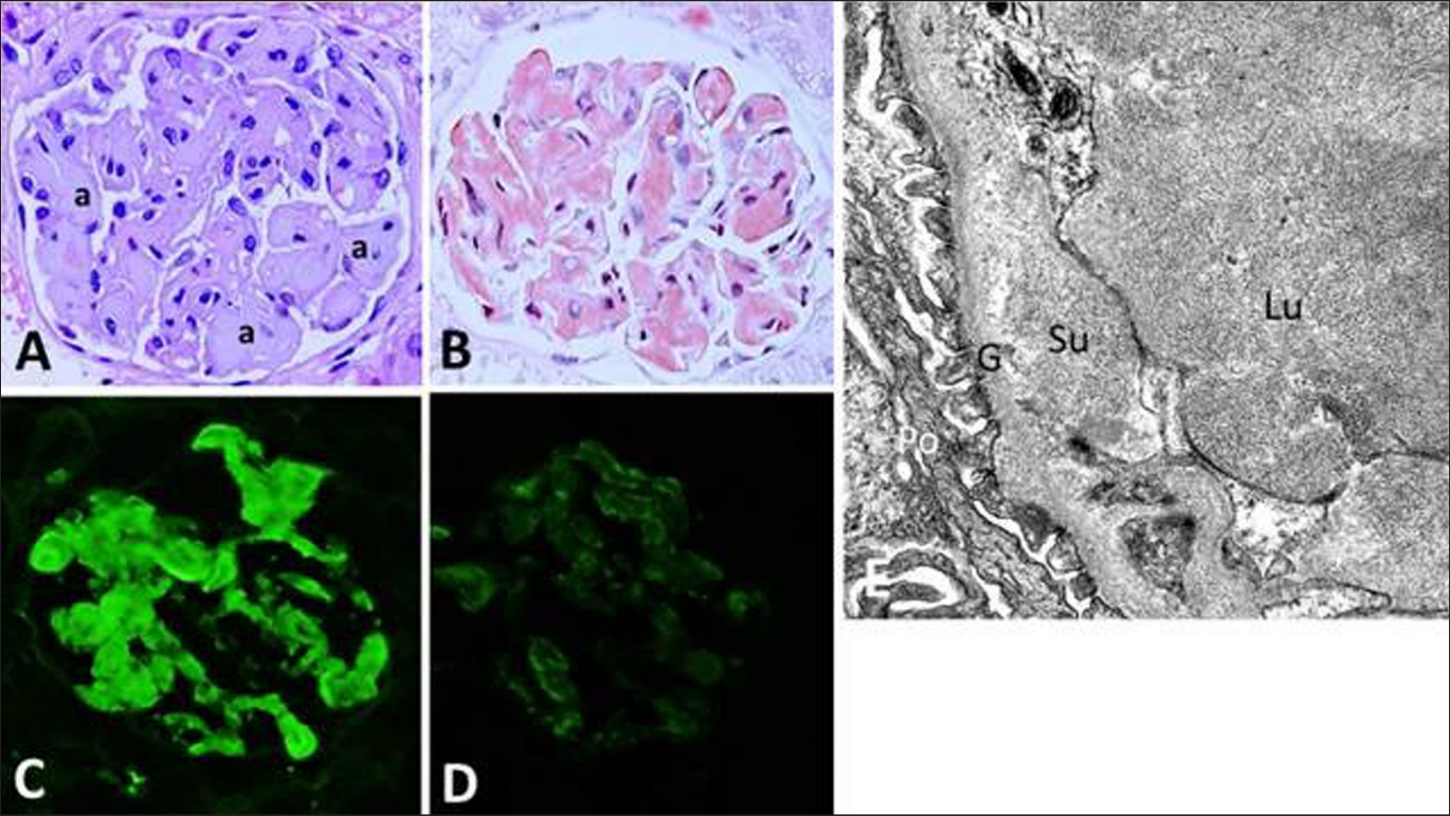
Renal amyloidosis. **(A)** Amyloid appearing as glassy material (a) deposited along glomerular capillary wall and lumens with hematoxylin &eosin stain, which **(B)** appears as “salmon-pink” deposits with Congo red stain. **(C)** The amyloid is composed of kappa light chain **(D)** but negative for lambda light chain, indicating light chain type of amyloid (×400 for panels A–D). **(E)** By electron microscopy (×15,000), amyloid appearing as short non-branching fibrils in the subendothelial location and glomerular capillary lumen. Su: subendothelial; Lu: glomerular capillary lumen; G: glomerular basement membrane; Po: podocyte

Longitudinal assessment of serum creatinine, serum electrolytes, and urinary protein and creatinine play a critical role in the care of patients with AL amyloidosis. Unlike cardiac involvement, kidney involvement does not markedly worsen survival unless the patient reaches end-stage kidney disease (ESKD) and the need for dialysis. An excellent recent prediction model for risk of dialysis has proven useful in planning treatment for these patients. Patients with an estimated glomerular filtration rate (eGFR) < 50 mL/min/1.73m^2^ and a 24-hour protein > 5 grams are predictors of ESKD. Patients with both of these parameters at diagnosis had a 3-year risk of ESKD of approximately 60% and 85% in two separate cohorts.^21^ In contrast, patients not having either of these parameters had a 3-year of ESKD of 0% and 4% in these cohorts. The reason is that the kidneys remove approximately 1 g of free light chains per day, so a B cell clone must produce amounts far greater than 1 gram per day to be pathologic to the kidneys. Deposition of light chains in the glomerular basement membrane induces proteinuria, which in turn is nephrotoxic (Figure 2). For this reason, when a patient presents as a medical mystery, screening for kappa and lambda light chains is a quick and effective way to help with early diagnosis and eventual prognostic risk stratification.^20^

### Step two: appropriate therapy for specific amyloid type

A detailed treatment plan for AL amyloidosis is beyond the scope of this review, so we refer to a recent consensus statement from the Mayo Clinic.^20^ In general, the treatment for AL amyloidosis is like that of multiple myeloma using melphalan and dexamethasone. Unique to AL amyloidosis is consideration for autologous stem cell transplantation. As an area of rapid change, it is imperative that a hematologist with expert experience be the team lead for this therapy. If secondary amyloidosis (AS) is discovered, then the underlying cause—end-stage renal disease (ESRD) infections—must be addressed for potential resolution. As mentioned, carpel tunnel syndrome has been identified as an early marker for both AL and ATTR and as a late finding in ESRD. It is now standard practice at Houston Methodist to send nerve tissue from carpel tunnel release surgery for staining for Congo and Texas red (Figure 1D, E).

### Step three: multidisciplinary longitudinal medical support for the patient

In addition to social work support to navigate coverage for chemotherapy, stem cell transplant, and solid organ transplant, psychological support is key for the patient and family. The cardiologist and the nephrologist must work together to approach volume overload in these patients. Diuretics and, if needed, dialysis are the backbone of therapy since beta blocker, angiotensin-converting enzyme inhibitors, angiotensin receptor blockers, and calcium channel blockers have caused heart block and hypotension in AL amyloidosis patients and are best avoided.^22^

A combination of loop diuretics and mineralocorticoid receptor antagonists is usually best for heart failure, nephrotic syndrome, and volume overload. Since beta blockers can cause bradycardia leading to pulseless electrical activity,^23^ amiodarone is often found to be helpful for rhythm control. Cardiac defibrillators remain controversial.^24^ Autonomic dysfunction in the form of orthostatic hypotension is very common in AL amyloidosis because of neuropathy involving small fibers. This can be treated symptomatically with multiple different agents. The most success has come from the use of midodrine and droxidopa.^25^ Doses as high as 20 mg three times a day may be needed for these patients. Patients should be advised to check blood pressure before laying down to avoid orthostatic hypertension.

The antibiotic doxycycline, studied in vitro and mouse models, has been found to disrupt and inhibit the formation of amyloid fibrils.^26^ This led to two retrospective studies looking at survival for doxycycline antibiotic prophylaxis in AL amyloidosis. In a study looking at survival in those patients receiving autologous stem cell transplantation, 455 patients were evaluated and 23% received oral doxycycline due to an oral penicillin allergy. Among the patients on doxycycline, the heart rate was significantly higher at 93%, indicating a survival advantage over the penicillin group with a heart rate at 59%.^27^

Solid organ transplantation is best pursued in very specialized organ transplant centers with the support of all necessary medical specialties who care for patients with AL amyloidosis. Before the new chemotherapeutic agents, heart transplantation was considered contraindicated. However, more recent data has shown great success in heart transplantation, even in the setting of delayed autologous stem cell transplant.^28^ In this series, 16 patients underwent heart transplant, including 2 combined heart kidney transplants for AL amyloidosis. Autologous stem cell transplant was performed in a total of 9 patients at a median of 13 months post-heart transplant. Survival was 87.5% at 1 year and 76.6% at 5 years, comparable to institutional outcomes for nonamyloid heart transplant recipients. The authors determined that a strategy of delayed autologous stem cell transplant 1-year post-heart transplant for patients with AL amyloidosis is feasible, safe, and associated with comparable outcomes to those undergoing an earlier autologous stem cell transplant strategy.

Kidney transplantation also has been successfully reported by several groups in the setting of AL amyloidosis. One study of 49 patients reported a graft survival of 94% at 1 year, 89% at 3 years, and 81% at 5 years. Those who had attained complete remission after therapy had longer graft survival than those with partial remission or no response.^29^ A second larger study included 75 patients showing 98.6% graft survival at 1 year, 97.7% graft survival at 3 years, and 85.5% graft survival at 10 years; similarly, those with better remission had better outcomes.^30^ It is the current standard of care in large centers to offer renal allografts to AL amyloidosis patients with ESKD who are already in complete remission or very good partial remission.

## Conclusion

While AL amyloidosis remains a challenging disease to treat, great strides have been made in improving long-term survival, both with chemotherapy and both stem cell and solid organ transplantation. The goal of this review was first and foremost to improve early recognition in order to improve outcomes of patients with AL amyloidosis. Care for this patient population cannot be done in a silo and, by design, must include a specialized amyloid center with a multidisciplinary team, including primary care, social work, psychology, neurology, gastroenterology, pulmonology, cardiology, nephrology, and most importantly the patient and their family.

## Key Points

- Bilateral carpel tunnel syndrome should now be considered an early warning signal of occult amyloidosis.
- Early and accurate diagnosis of the amyloid fibril type must always be done prior to initiation of any therapy.
- Solid organ transplantation is best pursued in very specialized organ transplant centers with the support of all necessary medical specialties who care for patients with immunoglobulin light chain amyloidosis.
- Prognosis is heavily dependent on the organ involved and plasma cell clone type.
